# Supplementary material for: Oxylipins Associated with D3-Creatine Muscle Mass/Weight and Physical Performance among Community-Dwelling Older Men
Source: Int J Mol Sci. 2022 Oct 25;23(21):12857. doi: 10.3390/ijms232112857 (PMC9655465; doi:10.3390/ijms232112857)
Supplement: Supplementary file 1 [file ijms-23-12857-s001.zip › ijms-1960277-supplementary.pdf]

Supplementary Information

Manuscript title: Oxylipins associated with D3-creatine muscle mass/weight and physical performance among community-dwelling older men

Figure S1. Flowchart from N=5994 men originally enrolled in MrOS to the analytic sample of n=463 men in the current report

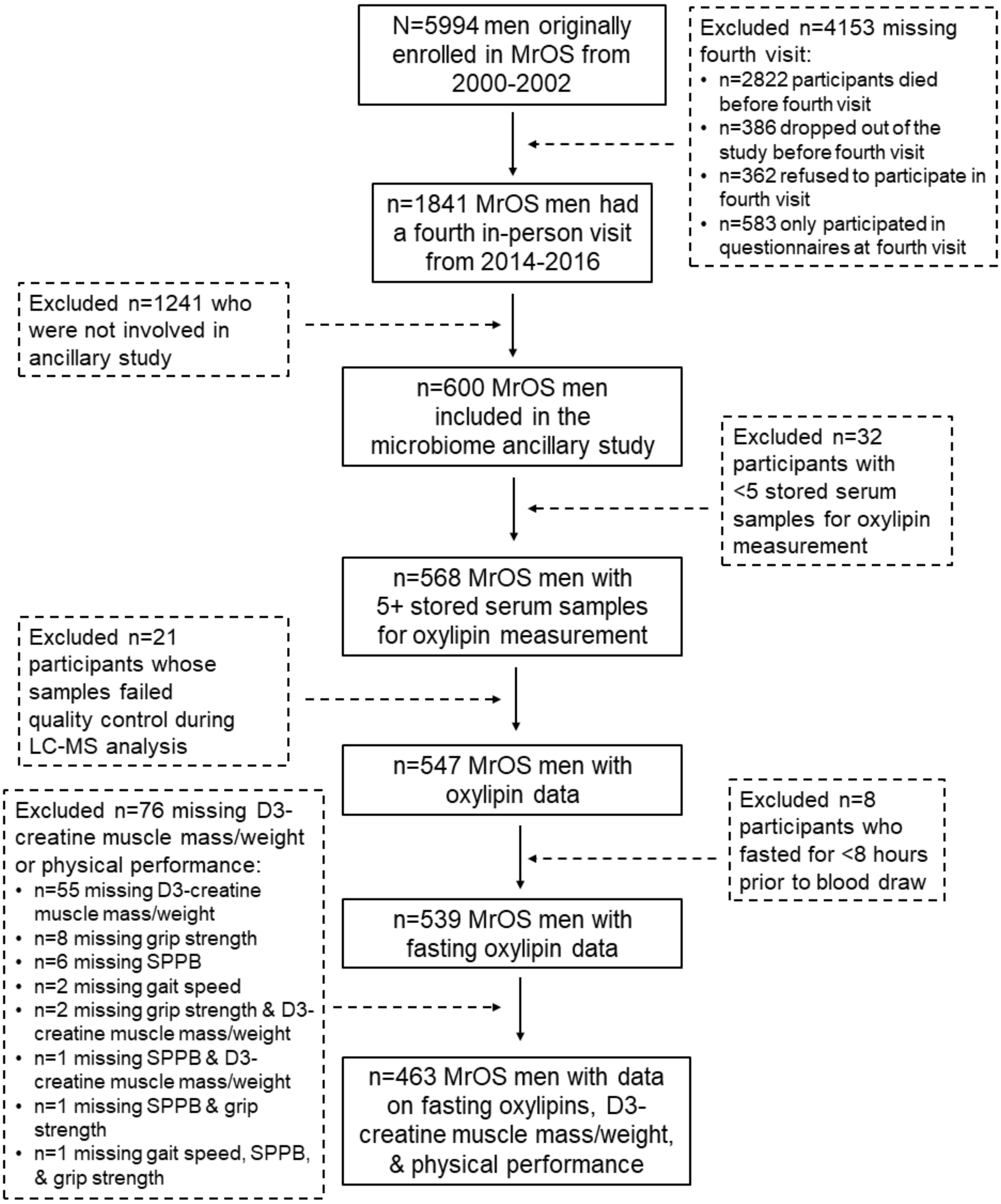

Table S1. Adjusted standardized beta coefficients between oxylipins and physical performance and D3-creatine muscle mass/weight, organized by fatty acid classification and most significant association with gait speed among 463 MrOs Men

Oxylipin:	Standardized beta coefficient (standard error), p-value, false discovery rate			
	D3-creatine Muscle Mass/weight	Gait Speed	Grip Strength	Short Physical Performance Battery
Fatty acid alcohol:				
14-HDoHE	0.14 (0.04), p=0.002, FDR=0.03	0.14 (0.04), p=0.002, FDR=0.03	0.02 (0.04), p=0.69, FDR=0.997	0.08 (0.04), p=0.06, FDR=0.23
4-HDoHE	0.13 (0.04), p=0.003, FDR=0.03	0.14 (0.04), p=0.001, FDR=0.03	0.03 (0.04), p=0.49, FDR=0.997	0.08 (0.04), p=0.08, FDR=0.27
12S-HEPE	0.10 (0.04), p=0.03, FDR=0.07	0.11 (0.04), p=0.01, FDR=0.06	-0.006 (0.04), p=0.9, FDR=0.997	0.05 (0.04), p=0.23, FDR=0.37
15-HETE	0.07 (0.04), p=0.11, FDR=0.18	0.13 (0.04), p=0.003, FDR=0.04	0.04 (0.04), p=0.37, FDR=0.997	0.07 (0.04), p=0.11, FDR=0.28
8S-HETE	0.09 (0.04), p=0.04, FDR=0.10	0.12 (0.04), p=0.005, FDR=0.05	0.01 (0.04), p=0.82, FDR=0.997	0.09 (0.04), p=0.04, FDR=0.16
11-HETE	0.10 (0.04), p=0.02, FDR=0.07	0.11 (0.04), p=0.009, FDR=0.05	0.001 (0.04), p=0.98, FDR=0.997	0.07 (0.04), p=0.10, FDR=0.27
9-HETE	0.09 (0.04), p=0.04, FDR=0.10	0.10 (0.04), p=0.02, FDR=0.06	0.02 (0.04), p=0.57, FDR=0.997	0.09 (0.04), p=0.04, FDR=0.16
9-HEPE	0.12 (0.04), p=0.005, FDR=0.03	0.09 (0.04), p=0.04, FDR=0.10	0.03 (0.04), p=0.56, FDR=0.997	0.03 (0.04), p=0.54, FDR=0.64
15-HEPE	0.12 (0.04), p=0.008, FDR=0.04	0.08 (0.04), p=0.05, FDR=0.11	-0.01 (0.04), p=0.74, FDR=0.997	0.03 (0.04), p=0.43, FDR=0.58
9-HODE	0.10 (0.04), p=0.02, FDR=0.07	0.08 (0.04), p=0.07, FDR=0.13	-0.02 (0.04), p=0.73, FDR=0.997	0.10 (0.04), p=0.02, FDR=0.13
5-HETE	0.07 (0.04), p=0.11, FDR=0.18	0.09 (0.04), p=0.03, FDR=0.08	0.02 (0.04), p=0.71, FDR=0.997	0.04 (0.04), p=0.34, FDR=0.47
12S-HETE	0.04 (0.04), p=0.39, FDR=0.46	0.07 (0.04), p=0.10, FDR=0.19	-0.006 (0.04), p=0.89, FDR=0.997	0.07 (0.04), p=0.10, FDR=0.27
13-HOTE	0.12 (0.04), p=0.006, FDR=0.03	0.08 (0.04), p=0.05, FDR=0.11	-0.0007 (0.04), p=0.99, FDR=0.997	0.14 (0.04), p=0.001, FDR=0.03
13S-HODE	0.12 (0.04), p=0.006, FDR=0.03	0.07 (0.04), p=0.10, FDR=0.18	0.04 (0.04), p=0.36, FDR=0.997	0.12 (0.04), p=0.007, FDR=0.10
17-HDoHE	0.09 (0.04), p=0.04, FDR=0.10	0.04 (0.04), p=0.31, FDR=0.41	0.02 (0.04), p=0.68, FDR=0.997	0.03 (0.04), p=0.54, FDR=0.64
9-HOTE	0.05 (0.04), p=0.22, FDR=0.28	0.02 (0.04), p=0.57, FDR=0.66	-0.02 (0.04), p=0.64, FDR=0.997	0.06 (0.04), p=0.19, FDR=0.34
Fatty acid diol:				
5,6-DiHETrE	0.10 (0.04), p=0.03, FDR=0.07	0.12 (0.04), p=0.004, FDR=0.04	0.02 (0.04), p=0.7, FDR=0.997	0.05 (0.04), p=0.23, FDR=0.37
6-trans-LTB4	0.07 (0.04), p=0.10, FDR=0.18	0.11 (0.04), p=0.01, FDR=0.06	-0.02 (0.04), p=0.61, FDR=0.997	0.06 (0.04), p=0.15, FDR=0.31
14,15-DiHETrE	-0.06 (0.04), p=0.17, FDR=0.23	-0.10 (0.04), p=0.02, FDR=0.07	0.04 (0.04), p=0.35, FDR=0.997	-0.05 (0.04), p=0.26, FDR=0.40
12,13-DiHOME	-0.002 (0.04), p=0.97, FDR=0.97	-0.09 (0.04), p=0.03, FDR=0.08	0.001 (0.04), p=0.97, FDR=0.997	-0.06 (0.04), p=0.14, FDR=0.30
15,16-DiHODE	-0.08 (0.04), p=0.06, FDR=0.12	-0.08 (0.04), p=0.05, FDR=0.11	0.02 (0.04), p=0.61, FDR=0.997	0.004 (0.04), p=0.93, FDR=0.94
9,10-DiHOME	0.06 (0.04), p=0.16, FDR=0.23	-0.09 (0.04), p=0.03, FDR=0.08	-0.02 (0.04), p=0.67, FDR=0.997	0.03 (0.04), p=0.52, FDR=0.64
11,12-DiHETrE	-0.03 (0.04), p=0.45, FDR=0.49	-0.06 (0.04), p=0.17, FDR=0.27	0.02 (0.04), p=0.7, FDR=0.997	0.003 (0.04), p=0.94, FDR=0.94
17,18-DiHETE	0.07 (0.05), p=0.13, FDR=0.19	0.02 (0.05), p=0.64, FDR=0.71	-0.02 (0.05), p=0.69, FDR=0.997	-0.01 (0.05), p=0.81, FDR=0.85
9,10-e-DiHO	0.03 (0.04), p=0.55, FDR=0.59	-0.05 (0.04), p=0.26, FDR=0.36	0.0001 (0.04), p=0.997, FDR=0.997	-0.03 (0.04), p=0.52, FDR=0.64
8,9-DiHETrE	-0.02 (0.04), p=0.62, FDR=0.65	-0.03 (0.04), p=0.52, FDR=0.64	0.003 (0.04), p=0.95, FDR=0.997	-0.04 (0.04), p=0.32, FDR=0.46
19,20-DiHDPE	0.16 (0.05), p=0.0005, FDR=0.02	0.01 (0.04), p=0.78, FDR=0.82	0.03 (0.04), p=0.48, FDR=0.997	0.06 (0.05), p=0.18, FDR=0.34
14,15-DiHETE	0.08 (0.05), p=0.10, FDR=0.18	-0.06 (0.05), p=0.23, FDR=0.34	0.002 (0.05), p=0.97, FDR=0.997	0.03 (0.05), p=0.55, FDR=0.64
9,10-DiHODE	-0.04 (0.04), p=0.41, FDR=0.46	0.02 (0.04), p=0.67, FDR=0.72	-0.07 (0.04), p=0.12, FDR=0.997	-0.01 (0.04), p=0.78, FDR=0.84
Fatty acid epoxide:				
9(10)-EpOME	0.08 (0.04), p=0.07, FDR=0.15	0.06 (0.04), p=0.13, FDR=0.22	-0.01 (0.04), p=0.78, FDR=0.997	0.10 (0.04), p=0.02, FDR=0.13
15,16-EpODE	0.06 (0.04), p=0.17, FDR=0.23	-0.07 (0.04), p=0.11, FDR=0.19	-0.04 (0.04), p=0.4, FDR=0.997	-0.02 (0.04), p=0.59, FDR=0.67
11,12-EpETrE	0.07 (0.04), p=0.10, FDR=0.18	0.05 (0.04), p=0.24, FDR=0.34	0.02 (0.04), p=0.64, FDR=0.997	0.05 (0.04), p=0.28, FDR=0.42
14,15-EpETrE	0.07 (0.04), p=0.11, FDR=0.18	0.03 (0.04), p=0.47, FDR=0.60	-0.01 (0.04), p=0.79, FDR=0.997	0.06 (0.04), p=0.16, FDR=0.32
alpha-9(10)-EpODE	0.05 (0.04), p=0.23, FDR=0.28	0.03 (0.04), p=0.55, FDR=0.66	-0.01 (0.04), p=0.81, FDR=0.997	0.06 (0.04), p=0.20, FDR=0.36
12(13)-EpOME	0.07 (0.04), p=0.12, FDR=0.18	0.02 (0.04), p=0.60, FDR=0.68	-0.05 (0.04), p=0.22, FDR=0.997	0.10 (0.04), p=0.02, FDR=0.13
alpha-12(13)-EpODE	0.10 (0.04), p=0.02, FDR=0.07	0.0005 (0.04), p=0.99, FDR=0.99	-0.007 (0.04), p=0.87, FDR=0.997	0.09 (0.04), p=0.03, FDR=0.15
9,10-EpO	0.01 (0.04), p=0.73, FDR=0.75	-0.008 (0.04), p=0.85, FDR=0.87	-0.03 (0.04), p=0.53, FDR=0.997	-0.02 (0.04), p=0.66, FDR=0.73
Fatty acid ketone:				
5-KETE	0.10 (0.04), p=0.02, FDR=0.07	0.12 (0.04), p=0.007, FDR=0.05	-0.004 (0.04), p=0.94, FDR=0.997	0.08 (0.04), p=0.08, FDR=0.27
9-KODE	0.13 (0.04), p=0.003, FDR=0.03	0.10 (0.04), p=0.02, FDR=0.06	-0.04 (0.04), p=0.33, FDR=0.997	0.14 (0.04), p=0.001, FDR=0.03
13-KODE	0.10 (0.04), p=0.02, FDR=0.07	0.09 (0.04), p=0.03, FDR=0.08	-0.01 (0.04), p=0.81, FDR=0.997	0.10 (0.04), p=0.02, FDR=0.14
Fatty acid triol:				
9,12,13-TriHOME	0.06 (0.04), p=0.17, FDR=0.23	0.05 (0.04), p=0.24, FDR=0.34	0.03 (0.04), p=0.55, FDR=0.997	0.07 (0.04), p=0.09, FDR=0.27
Nitro-fatty acid:				
10-Nitrooleate	-0.05 (0.04), p=0.27, FDR=0.33	-0.05 (0.04), p=0.21, FDR=0.32	0.009 (0.04), p=0.84, FDR=0.997	-0.07 (0.04), p=0.12, FDR=0.28

Models were adjusted for age, more than high school education, Physical Activity Scale for the Elderly, western style dietary pattern score, and fish oil supplement use.
Gray shaded cells: associations with p<0.05 & FDR≤10%.

Table S2. Fatty acid classification, abbreviation, and full name of the 42 oxylipins included in this report

**Fatty acid classification:**	**Polyunsaturated fatty acid type oxylipin is derived from:**	**Oxylipin abbreviation**	**Oxylipin**	**Number (%) of participants oxylipin was detected in**
Fatty acid alcohol	Omega-3	14-HDoHE	14-hydroxydocosa-4,7,10,12,16,19-hexaenoic acid	442 (95%)
Fatty acid alcohol	Omega-3	4-HDoHE	4-hydroxydocosa-5,7,10,13,16,19-hexaenoic acid	452 (98%)
Fatty acid alcohol	Omega-3	12S-HEPE	12-Hydroxy-5,8,10,14,17-eicosapentaenoic acid	457 (99%)
Fatty acid alcohol	Omega-6	15-HETE	15-hydroxyeicosa-5,8,11,13-tetraenoic acid	460 (99%)
Fatty acid alcohol	Omega-6	8S-HETE	8-hydroxyeicosa-5,9,11,14-tetraenoic acid	418 (90%)
Fatty acid alcohol	Omega-6	11-HETE	11-Hydroxy-arachidonic acid	461 (100%)
Fatty acid alcohol	Omega-6	9-HETE	9-hydroxyeicosa-5,7,11,14-tetraenoic acid	436 (94%)
Fatty acid alcohol	Omega-6	9-HEPE	9-Hydroxy-5,7,11,14,17-icosapentaenoic acid	455 (98%)
Fatty acid alcohol	Omega-6	15-HEPE	15-hydroxyeicosa-5,8,11,13,17-pentaenoic acid	454 (98%)
Fatty acid alcohol	Omega-6	9-HODE	9-Hydroxylinoleic acid	463 (100%)
Fatty acid alcohol	Omega-6	5-HETE	5-Hydroxy-6,8,11,14-eicosatetraenoic acid	460 (99%)
Fatty acid alcohol	Omega-6	12S-HETE	12-Hydroxy-5,8,10,14-eicosatetraenoic acid	461 (100%)
Fatty acid alcohol	Omega-3	13-HOTE	13-hydroxyoctadeca-9,11,15-trienoic acid	460 (99%)
Fatty acid alcohol	Omega-6	13S-HODE	13-Hydroxyoctadecadienoic acid	463 (100%)
Fatty acid alcohol	Omega-3	17-HDoHE	17-hydroxy-4,7,10,13,15,19-docosahexaenoic acid	460 (99%)
Fatty acid alcohol	Omega-3	9-HOTE	9-hydroxyoctadeca-10,12,15-trienoic acid	463 (100%)
Fatty acid diol	Omega-6	5,6-DiHETrE	5,6-dihydroxyeicosa-8,11,14-trienoic acid	460 (99%)
Fatty acid diol	Omega-6	6-trans-LTB4	6-trans-Leukotriene B4	382 (83%)
Fatty acid diol	Omega-6	14,15-DiHETrE	14,15-dihydroxyeicosa-5,8,11-trienoic acid	460 (99%)
Fatty acid diol	Omega-6	12,13-DiHOME	12,13-dihydroxyoctadec-9-enoic acid	463 (100%)
Fatty acid diol	Omega-3	15,16-DiHODE	15,16-dihydroxyoctadeca-9,12-dienoic acid	460 (99%)
Fatty acid diol	Omega-6	9,10-DiHOME	9,10-dihydroxyoctadec-12-enoic acid	461 (100%)
Fatty acid diol	Omega-6	11,12-DiHETrE	11,12-Dihydroxyicosa-5,8,14-trienoic acid	460 (99%)
Fatty acid diol	Omega-3	17,18-DiHETE	17,18-dihydroxyeicosa-5,8,11,14-tetraenoic acid	460 (99%)
Fatty acid diol	-----	9,10-e-DiHO	9S,10R-dihydroxy-stearic acid	463 (100%)
Fatty acid diol	Omega-6	8,9-DiHETrE	8,9-dihydroxyeicosa-5,11,14-trienoic acid	457 (99%)
Fatty acid diol	Omega-3	19,20-DiHDPE	19,20-dihydroxydocosa-4,7,10,13,16-pentaenoic acid	460 (99%)
Fatty acid diol	Omega-3	14,15-DiHETE	14,15-dihydroxyeicosa-5,8,11,17-tetraenoic acid	405 (87%)
Fatty acid diol	Omega-3	9,10-DiHODE	9,10-dihydroxyoctadeca-12,15-dienoic acid	441 (95%)
Fatty acid epoxide	Omega-6	9(10)-EpOME	9(10)-epoxy-12Z-octadecenoic acid	461 (100%)
Fatty acid epoxide	Omega-3	15,16-EpODE	15(16)-epoxy-9,12-octadecadienoic acid	452 (98%)
Fatty acid epoxide	Omega-6	11,12-EpETrE	11,12-Epoxyeicosa-5,8,14-trienoic acid	439 (95%)
Fatty acid epoxide	Omega-6	14,15-EpETrE	11-Hydroxy-14,15-epoxyeicosatrienoic acid	419 (90%)
Fatty acid epoxide	Omega-3	alpha-9(10)-EpODE	9(10)-epoxy-12,15-octadecadienoic acid	415 (90%)
Fatty acid epoxide	Omega-6	12(13)-EpOME	12,13-epoxy-9-octadecenoic acid	459 (99%)
Fatty acid epoxide	Omega-3	alpha-12(13)-EpODE	12(13)-epoxy-9,15-octadecadienoic acid	444 (96%)
Fatty acid epoxide	-----	9,10-EpO	9,10-Epoxystearic acid	454 (98%)
Fatty acid ketone	Omega-3	5-KETE	5-ketoeicosa-6,8,11,14-tetraenoic acid	392 (85%)
Fatty acid ketone	Omega-6	9-KODE	9-ketooctadeca-10,12-dienoic acid	461 (100%)
Fatty acid ketone	Omega-6	13-KODE	13-ketooctadeca-9,11-dienoic acid	456 (98%)
Fatty acid triol	Omega-6	9,12,13-TriHOME	9,12,13-trihydroxyoctadec-10-enoic acid	461 (100%)
Nitro-fatty acid	Omega-9	10-Nitrooleate	10-nitrooleic acid	373 (81%)

Table S3a. Attenuation in associations between select oxylipins and gait speed after additionally adjusting for D3-creatine muscle mass/weight among 463 MrOs Men

	**Model 1*:** Association between oxylipins *or* D3-creatine muscle mass/weight with respect to gait speed	**Model 2*:** Association between oxylipins and D3-creatine muscle mass/weight	**Model 3**:** Associations between oxylipins or D3-creatine muscle mass/weight with respect to gait speed, while further adjusting for oxylipin and D3-creatine muscle mass/weight in the same model			
			Oxylipin		D3-creatine muscle mass/weight	
	Standardized beta coefficient (standard error), p-value	Standardized beta coefficient (standard error), p-value	Standardized beta coefficient (standard error), p-value	Attenuation ****	Standardized beta coefficient (standard error), p-value	Attenuation ****
**D3-creatine Muscle Mass/weight**	-0.30 (0.10), p=0.003	-----	-----			
**Oxylipins***:**						
Fatty acid alcohol:						
14-HDoHE	0.14 (0.04), p=0.002	0.14 (0.04), p=0.002	0.10 (0.04), p=0.02	28%	0.28 (0.04), p<0.0001	5%
4-HDoHE	0.14 (0.04), p=0.001	0.13 (0.04), p=0.003	0.10 (0.04), p=0.01	26%	0.28 (0.04), p<0.0001	5%
12S-HEPE	0.11 (0.04), p=0.01	0.10 (0.04), p=0.03	0.08 (0.04), p=0.05	25%	0.28 (0.04), p<0.0001	3%
15-HETE	0.13 (0.04), p=0.003	0.07 (0.04), p=0.11	-----	-----	-----	-----
8S-HETE	0.12 (0.04), p=0.005	0.09 (0.04), p=0.04	0.09 (0.04), p=0.02	22%	0.28 (0.04), p<0.0001	3%
11-HETE	0.11 (0.04), p=0.009	0.10 (0.04), p=0.02	0.08 (0.04), p=0.04	25%	0.28 (0.04), p<0.0001	3%
9-HETE	0.10 (0.04), p=0.02	0.09 (0.04), p=0.04	0.08 (0.04), p=0.06	24%	0.28 (0.04), p<0.0001	3%
9-HEPE	0.09 (0.04), p=0.04	0.12 (0.04), p=0.005	0.05 (0.04), p=0.20	40%	0.29 (0.04), p<0.0001	3%
5-HETE	0.09 (0.04), p=0.03	0.07 (0.04), p=0.11	-----	-----	-----	-----
Fatty acid diol:						
5,6-DiHETrE	0.12 (0.04), p=0.004	0.10 (0.04), p=0.03	0.10 (0.04), p=0.02	22%	0.28 (0.04), p<0.0001	4%
6-trans-LTB4	0.11 (0.04), p=0.01	0.07 (0.04), p=0.10	-----	-----	-----	-----
14,15-DiHETrE	-0.10 (0.04), p=0.02	-0.06 (0.04), p=0.17	-----	-----	-----	-----
12,13-DiHOME	-0.09 (0.04), p=0.03	-0.002 (0.04), p=0.97	-----	-----	-----	-----
9,10-DiHOME	-0.09 (0.04), p=0.03	0.06 (0.04), p=0.16	-----	-----	-----	-----
Fatty acid ketone:						
5-KETE	0.12 (0.04), p=0.007	0.10 (0.04), p=0.02	0.09 (0.04), p=0.04	25%	0.28 (0.04), p<0.0001	3%
9-KODE	0.10 (0.04), p=0.02	0.13 (0.04), p=0.003	0.07 (0.04), p=0.10	35%	0.28 (0.04), p<0.0001	3%
13-KODE	0.09 (0.04), p=0.03	0.10 (0.04), p=0.02	0.06 (0.04), p=0.13	32%	0.29 (0.04), p<0.0001	2%

*Models 1 & 2: Adjusted for age, more than high school education, Physical Activity Scale for the Elderly, western style dietary pattern score, and fish oil supplement use.
**Model 3: Adjusted for D3-creatine muscle mass/weight and an oxylipin in the same model, in addition to age, more than high school education, Physical Activity Scale for the Elderly, and western style dietary pattern score.
***Select oxylipins included are the subset that were associated with gait speed at a p<0.05.
Note, all continuous variables were standardized to a mean of zero and standard deviation of one.
****Attenuation=100*(beta coefficient from initial model – beta coefficient from further adjusted model) / beta coefficient from initial model).

Table S3b. Attenuation in associations between select oxylipins and short physical performance battery (SPPB) after additionally adjusting for D3-creatine muscle mass/weight among 463 MrOs Men

	**Model 1*:** Association between oxylipins *or* D3-creatine muscle mass/weight with respect to SPPB	**Model 2*:** Association between oxylipins and D3-creatine muscle mass/weight	**Model 3**:** Associations between oxylipins or D3-creatine muscle mass/weight with respect to SPPB, while further adjusting for oxylipin and D3-creatine muscle mass/weight in the same model			
			Oxylipin		D3-creatine muscle mass/weight	
	Standardized beta coefficient (standard error), p-value	Standardized beta coefficient (standard error), p-value	Standardized beta coefficient (standard error), p-value	Attenuation ****	Standardized beta coefficient (standard error), p-value	Attenuation ****
**D3-creatine Muscle Mass/weight**	0.33 (0.04), p<0.0001	-----	-----			
**Oxylipins***:**						
Fatty acid alcohol: 13-HOTE	0.14 (0.04), p=0.001	0.12 (0.04) p=0.006	0.10 (0.04) p=0.02	28%	0.31 (0.04) p<0.0001	4%
13S-HODE	0.12 (0.04), p=0.007	0.12 (0.04) p=0.006	0.08 (0.04) p=0.06	33%	0.32 (0.04) p<0.0001	3%
Fatty acid ketone: 9-KODE	0.14 (0.04), p=0.001	0.13 (0.04) p=0.003	0.10 (0.04) p=0.02	29%	0.31 (0.04) p<0.0001	4%

*Models 1 & 2: Adjusted for age, more than high school education, Physical Activity Scale for the Elderly score, western style dietary pattern score, and fish oil supplement use.
**Model 3: Adjusted for D3-creatine muscle mass/weight and an oxylipin in the same model, in addition to age, more than high school education, Physical Activity Scale for the Elderly, and western style dietary pattern score.
***Select oxylipins included are the subset that were associated with SPPB at a p<0.05.
Note, all continuous variables were standardized to a mean of zero and standard deviation of one.
****Attenuation=100*(beta coefficient from initial model – beta coefficient from further adjusted model) / beta coefficient from initial model).

Table S4. Fatty acid classification, abbreviation, and full name of the 34 oxylipins excluded from this report because they were detected in less than 80% of the cohort

**Fatty acid classification:**	**Oxylipin abbreviation**	**Oxylipin**	**Number (%) of participants oxylipin was detected in**
Fatty acid alcohol	5S-HEPE	5-Hydroxy-6,8,11,14,17-eicosapentaenoic acid	347 (75%)
Fatty acid alcohol	20-HETE	20-Hydroxyarachidonic acid	152 (33%)
Fatty acid diol	LTB4	Leukotriene B4	325 (70%)
Fatty acid diol	5S,15R-diHETE	5,15-dihydroxyeicosa-6,8,11,13-tetraenoic acid	290 (63%)
Fatty acid diol	8,15-DiHETE	8,15-dihydroxyeicosa-5,9,11,13-tetraenoic acid	271 (59%)
Fatty acid diol	LTB5	Leukotriene B5	105 (23%)
Fatty acid diol	12,13-DiHODE	12,13-dihydroxyoctadeca-9,15-dienoic acid	70 (15%)
Fatty acid epoxide	12(13)Ep-9-KODE	trans-12,13-epoxy-11-oxo-trans-9-octadecenoic acid	330 (71%)
Fatty acid epoxide	17(18)-EpETE	17,18-Epoxy-5,8,11,14-eicosatetraenoic acid	309 (67%)
Fatty acid epoxide	11(12)-EpETE	11(12)-epoxy-5,8,14,17- eicosatetraenoic acid	247 (53%)
Fatty acid epoxide	19(20)-EpDPE	18-(3-ethyloxiran-2-yl)octadeca-4,7,10,13,16-pentaenoic acid	199 (43%)
Fatty acid epoxide	8(9)-EpETrE	8,9-Epoxyeicosa-5,11,14-trienoic acid	190 (41%)
Fatty acid epoxide	14(15)-EpETE	14(15)-epoxy-5,8,11,17-eicosatetraenoic acid	131 (28%)
Fatty acid epoxide	16(17)-EpDPE	16(17)-epoxy-4,7,10,13,19-docosapentaenoic acid	59 (13%)
Fatty acid ketone	15-KETE	15-ketoeicosa-5,8,11,13-tetraenoic acid	273 (59%)
Fatty acid triol	PGF3alpha	9,11,15-trihydroxy-5,13,1Z-prostatrienoic acid	104 (22%)
Fatty acid triol	Lipoxin A4	5,6,15-trihydroxyeicosa-7,9,11,13-tetraenoic acid	0
Fatty acid triol	Resolvin D1	Resolvin D1	0
Nitro-fatty acid	10-Nitrolinoleate	10-nitrolinoleic acid	66 (14%)
Nitro-fatty acid	9-Nitrooleate	9-nitrooleic acid	40 (9%)
Prostanoid	PGF2alpha	Prostaglandin F2a	243 (52%)
Prostanoid	PGE2	Prostaglandin E2	162 (35%)
Prostanoid	PGD2	Prostaglandin D2	160 (35%)
Prostanoid	PGE3	Prostaglandin E3	101 (22%)
Prostanoid	PGE1	Prostaglandin E1	73 (16%)
Prostanoid	15-deoxy PGJ2	15-Deoxy-delta-12,14-Prostaglandin J2	34 (7%)
Prostanoid	15-Keto PGE2	15-Keto-prostaglandin E2	14 (3%)
Prostanoid	6-keto-PGF1alpha	6-Ketoprostaglandin F1 alpha	0
Thromboid	TXB2	Thromboxane B2	322 (70%)
Fatty acid hydroperoxide	12-HpETE	12-hydroperoxyicosa-5,8,10,14-tetraenoic acid	0
Fatty acid hydroperoxide	13-HpODE	13-hydroperoxyoctadeca-9,11-dienoic acid	0
Fatty acid hydroperoxide	15-HpETE	15-hydroperoxyeicosa-5,8,11,13-tetraenoic acid	0
Fatty acid hydroperoxide	5-HpETE	5-hydroperoxyeicosa-6,8,11,14-tetraenoic acid	0
Fatty acid hydroperoxide	9-HpODE	9-hydroperoxyoctadeca-10,12-dienoic acid	0
